# Self-Formation of Nanoporous Metal–Organic Framework/Water Interphase

**DOI:** 10.1021/acsanm.5c05761

**Published:** 2026-03-31

**Authors:** Jaime Gonzalez-Gomez, Salvador R.-G. Balestra, Alessandro Siria, Pilar Aranda, Javier Perez-Carvajal

**Affiliations:** † Instituto de Ciencia de Materiales de Madrid CSIC, Madrid 28049, Spain; ‡ Departamento de Física Atómica, Molecular y Nuclear, Área de Física Teórica, Universidad de Sevilla, Sevilla 41012, Spain; § Laboratoire de Physique de l’Ecole Normale Superieure, ENS, Université PSL, CNRS, Sorbonne Universite, Universite de Paris, Paris 75005, France; ∥ Center for Advanced Nanoscale Functionalites, College of Physics and Optoelectronic Enginneering, Shenzhen Univeristy, Shenzhen 518060, China; ⊥ Department of mechanical engineering, Tsinghua University, Beijing 100190, China

**Keywords:** metal−organic frameworks, Zeolitic imidazole framework, nanoparticles, water−air interphase, pollutants

## Abstract

The reduction of the energy cost of environmental remediation processes is one of the major issues that afford modern societies. Colloidal nanoparticles of molecular sieve-type materials are widely used as adsorbents and catalysts in separation, conversion, and recovery processes, but still show limitations during their recovery due to their nano size, which hinders the viability of those materials in large-scale processes. Herein, we propose a simple alternative method to induce the formation of self-organized floating metal–organic framework (MOF) nanoparticles into films. These films are confined regions formed by entrapped MOF particles between 500 nm and microns morphologies over the liquid water. This feature will allow for low energy consumption recovery of the nanoparticles at the industrial level. Furthermore, functional MOF particles can remove dyes from water media and promote discoloration, in the case of methylene blue.

## Graphical abstract

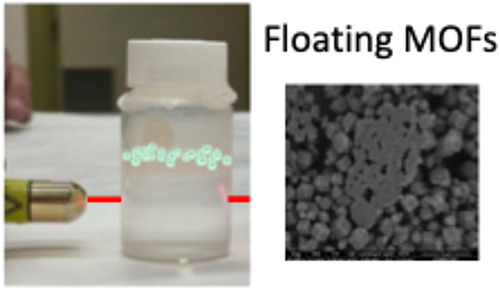

## 1 Introduction

The development of functional nanoparticles in stable suspension, such as colloids,^1^ has attracted large interest in material science since these active particles present the possibility of flowing as a liquid and therefore, they can be easily incorporated in industrial processes with high homogeneity. The preparation of particles in the nanorange of sizes that present colloidal stability has been also targeted to obtain deposited and conformed materials, such as films or coatings,^2^ where continuity is required. Colloids have been directly used in liquid media for certain chemical processes as adsorbents or catalysts^3^ with certain advantages from their equivalent non-colloidal phases.

Different methodologies can be found in literature to prepare colloidal nanoparticles within a large variety of chemical compositions and structures, like SiO_2_ and metal oxide nanoparticles,^4^ polymers,^5^ alloys, and metal–organic framework (MOF).^6–8^ MOFs are crystalline porous solids used in countless applications, including surface-related applications, like gas storage and separation, catalysis, drug delivery, or photonics.^9^ The specific case of nanoMOFs enhances its applications and functionalities, including intravenous drug delivery, bioimaging, nanomotors, and processing, among others.^10^ Zeolitic imidazole frameworks (ZIF) are a classic subfamily of MOF that presents the crystalline topology of zeolites, characterized by large cages and small apertures. Among this large subfamily of MOF, it highlights the so-called ZIF-8, which was one of the first MOF examples exhibiting high thermal and chemical stability.^11,12^ ZIF-8, a sodalite-type structure raised by the combination of Zn (II) ions and 2-methylimidazolate organic molecules, presents a permanent porosity with a large surface area, typically over 1200 m^2^ g^–1^, crystalline-to-crystalline transitions, and low water affinity, even at high relative-humidity conditions, so this material is suitable for being used in CO_2_ sensing under humid conditions,^13^ among other applications, including gas separation. Moreover, its high affinity toward organophilic compounds even in the presence of water^8^ raises ZIF-8 as a very good candidate for water remediation of organic pollutants.^14^ The control of crystal size and shape^15^ has opened routes toward the preparation of solid superstructures formed by the arrangement of ZIF-8 nanoparticles by ice templating,^16^ spray dryer,^17^ or evaporation-induced self-assembly,^8^ among others. Other ZIFs, like ZIF-67 and its derivatives, have been largely explored, especially related to catalytic processes.^17–19^

In biology, a floating film is defined as a unique configuration wherein *particles behave as a fluid surface instead of a rigid one*. The obtention of some of those kinds of floating systems is especially relevant in separation when dealing with water surface contaminants, like oils or emulsions,^20,21^ or in the pathogen growth control on stagnant water.^22,23^

The preparation of floating films can be easily accessed by using processed polymers. In general, two strategies can be followed to produce floating films: the so-called “lotus effect” approach, in which the solid surface promotes air trapping with the consequent increase in the apparent contact angle,^24^ and hydrophobic particles, such as in the case of liquid marbles.^25^ Previous strategies to prepare MOF systems that can be easily recovered, like ice-templated superstructures^26^ or surfactant-induced MOF films,^27^ have been explored. MOFs present the advantage compared with established remediation materials, such as polymer membranes, of their high surface area (typically over 1000 m^2^/g), comparable to the best activated carbons (AC); in addition, ZIF-8 can be prepared in water, in a short time, and at room temperature; meanwhile, AC typically requires 1000 °C in their synthesis procedures.

Herein, we report a simple method to induce the formation of a confined region that behaves as a floating film formed by water and entrapped MOF particles ranging from nanometer to micrometer sizes with different morphologies. Then, the rationalization of some parameter that rules the phenomena and, finally, the exploration of its potential applications. We will show how the MOF particles in this singular configuration can adsorb highly conjugated organic molecules insoluble in water and set them at the water/air interphases. Furthermore, how the MOF particles surprisingly decompose catalytically the model molecules methylene blue after its adsorption.

## 2 Experimental Section

### 2.1 Materials

All chemical reagents and solvents were purchased from Sigma-Aldrich and used as received without further purification. Ultrapure water (resistivity of 18.2 MΩ cm) was obtained with a Purelab Chorus 1 instrument from Elga.

### 2.2 Synthesis of ZIF-8 TRD Particles

In a typical synthesis of a truncated dodecahedron of ZIF-8 (ZIF-8-TRD), we followed a slightly modified protocol previously reported by Cravillon et al.^12^ 0.3 g (1.37 mmol) of Zn­(CH_3_COO)_2_·2H_2_O (99.9%) is dissolved in 5 mL of water and mixed with 2-methylimidazole (1.1 g) previously dissolved in 6.5 mL of water. The resulting mixture was then stirred over 600 rpm at room temperature for a variable time, ranging from 5 s to 10 min (5 s (ZIF-8–100), 2 min (ZIF-8–250), 5 min (ZIF-8–470), 10 min ZIF-8-TRD). Actually, the initial mixture became turbid after some seconds, but the reaction was allowed to progress for a variable time.

### 2.3 Synthesis of ZIF-8 CB Particles

In a typical synthesis of a cubic ZIF-8 (ZIF-8-CB), 0.3 g of Zn­(CH_3_COO)_2_·2H_2_O (99.9%) was dissolved in 5 mL of water, and then 2-methylimidazole (1.1 g) dissolved in 6.5 mL of water was added. In the present case, the resulting mixture was stirred below 100 rpm at room temperature for 30 min.

### 2.4 Synthesis of ZIF-8 RD Particles

For the synthesis of rhombic dodecahedra ZIF-8 (ZIF-8-RD) particles, 0.3 g of Zn­(CH_3_COO)_2_·2H_2_O (99.9%) was dissolved in 5 mL of water and mixed with 1.1 g of 2-methylimidazole dissolved in 6.5 mL while keeping the mixture under gentle stirring at room temperature for 24–96 h.

### 2.5 Synthesis of ZIF-8-Cl and ZIF-8-NO3 Particles

ZIF-8-Cl and ZIF-8-NO3 are prepared following the same procedure described for ZIF-8-TRD (10 min) but exchanging Zn­(CH_3_COO)_2_·2H_2_O reagent with 1.37 mmol of ZnCl_2_ or Zn­(NO_3_)_2_, respectively.

### 2.6 Purification and Extraction of the ZIF-8 Materials

Once the set time for stirring had been achieved, the MOF-formed nanoparticles were separated from the solvent by centrifugation at 12,000 rpm for 15 min using 50 mL conical tubes. The recovered precipitates were then redispersed in 15 mL of water twice, recovering the particles each time by centrifugation at 12,000 rpm (AFI, model LISA with ventilation).

### 2.7 Preparation of ZIF-8 Floating Interphase

ZIF-8 floating interphases have been formed by dispersing the particles ranging from 1 to 8 mg/mL ZIF-8 in 15 mL of water. The dispersion is dropped in a glass vial, and the system is left to evolve over time until the floating films are formed in the 14 experiments performed.

### 2.8 Characterization Techniques

X-ray powder diffraction (PXRD) of the prepared ZIF solids was recorded on a powder X-ray diffraction (Bruker D8 Advance diffractometer (DAVINCI), working with λ Cu = 1.5406 Å) from 5 to 40° (2θ). Field-emission scanning electron microscopy (FE-SEM) images were collected in a FEI-NOVA NanoSEM 230 microscope with an acceleration voltage of 2 kV. The size of crystals was calculated from FE-SEM images by averaging the size of crystals observed in images of different areas of the sample. Volumetric N_2_ sorption measurements were collected at 77 K using ASAP 2010 and TriStar sorption system (Micromeritics). Before the adsorption measurements, the samples were outgassed at 120 °C overnight. IR spectra of membranes were obtained using an attenuated total reflectance IR microscope coupled to a Vertex 70v Spectrophotometer (Bruker). Contact angle measurement was determined using a 7 mL drop of water deposited on top of a pellet of ZIF-8 samples, previously obtained by pressing the ZIF-8 powder at 4 tons for 4 s. The molecules derived from methylene blue (10^–3^ M as starting concentration) in the total decolorated media have been analyzed by a liquid chromatograph that is coupled to a quadrupole ion trap mass spectrometer with ESI and APCI sources (Thermo Fisher).

## 3 Results and Discussion

The crystalline structure of ZIF-8 is formed by coordination bonds between Zn metal nodes and N atoms from the imidazole heterocycle molecules, raising a periodic 3D structure that presents a permanent porosity due to its structural cavities of typically 1.1 nm diameter (Figure 1a). ZIF-8 particles were grown in close to sustainable conditions, using water as the synthesis media, at room temperature. ZIF-8 was selected among the large chemical composition diversity of MOF since it is based on Zn atoms, which is an abundant element in the earth, presents low toxicity and higher thermal stability than other MOF structures.

**Figure 1:**
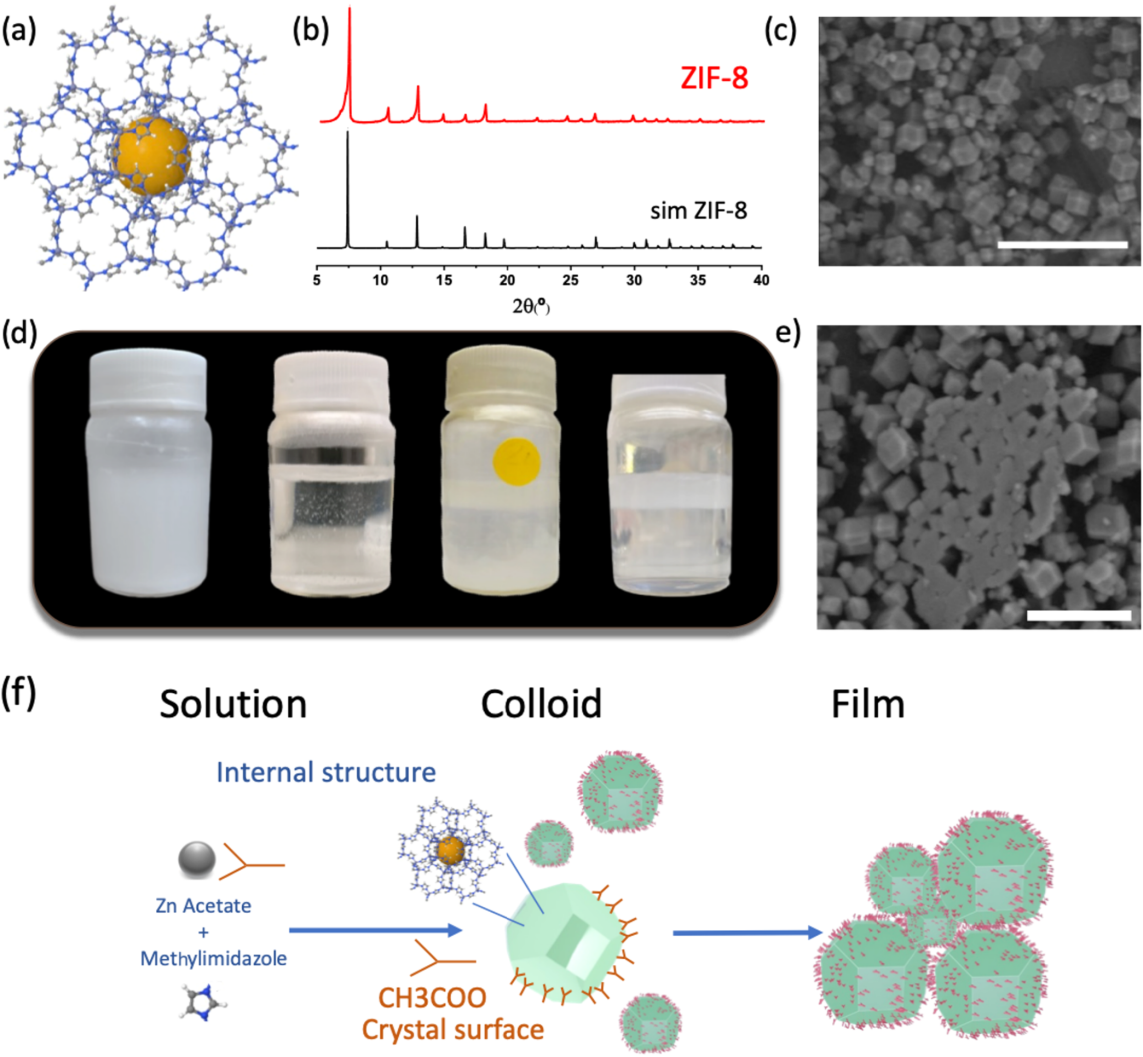
Scheme of the atomic structure ZIF-8 metal–organic framework (a), PXRD of prepared ZIF-8 particles (up) in addition to simulated ZIF-8 powder pattern (down) (b), FE-SEM images of dried crystals of ZIF-8 (c), pictures in function of time of redispersed ZIF-8 particles and its regional confinement evolution to form the floating film (time evolving from left to right) (d), and representative FE-SEM images of aggregates collected from the formed film (e). Scale bar in (c) and (e) subfigures are 5 and 3 μm, respectively. (f) Schematic representation of the system (structure/chemical composition), synthesis, and particle evolution to floating film.

The success of the synthetic approach followed to prepare ZIF-8 (Figure 1a) crystalline particles (ZIF-8-RD) is corroborated by the comparison of the XRD powder pattern (Figure 1b) of the dry solid obtained and the theoretical pattern simulated from crystalline data (CSD). XRD diffractograms show the presence of the expected crystalline phase in all of the prepared solids^13^ where the typical planes related to (110), (200), (211) are clearly identified at 7.3, 10.3, and 12.7 in 2 theta angles, respectively. The determined permanent porosity of the thermal activated solids is in good agreement with previously reported data (1299 m^2^/g^–1^) (Figure S1), and the observation of formed ZIF-8 particles by FE-SEM confirms a typical formation of rhombic dodecahedra (RD) crystals (Figure 1c) with a main size of 1200 nm (Figure S2).

Interestingly, a redispersion of those RD crystals in water in a ratio of 5 mg mL^–1^ gives a dispersion of ZIF-8 particles that evolves during time under static conditions to a floating interphase after 6 days (Figure 1d). The observed phenomenon is produced as follows: (1) an initial stage where the particles remain dispersed in the water media as a suspension, typically it lasts for a few hours; (2) in a second stage the particles fall to the bottom of the vial while some large aggregates are suspended inside the bulk of the liquid phase; (3) stage in which initially is observed a floating region in the limiting interphase between liquid and air that evolves as aggregates and particles move from the bottom to the floating region, and finally, (4) formation of a floating interphase by MOF particles on water that remains stable for a variable time, typically during two months to over a year. To establish the end of the formation phenomena, a laser (Figure 2a) was pointed to the bulk of water obtaining coherence, which indicates a low to none quantity of particles dispersed in the bulk liquid phase. For comparison, the laser was allowed to cross through a second vial that contains a fresh dispersion of ZIF-8 colloidal nanoparticles showing the expected dispersion in the wave. When the laser is focused on the floating film region (Figure 2b), a clear pink color is observed due to the presence of the particles confined in a certain region. To further corroborate that the formed floating film is actually composed of the mentioned particles, the film is observed by recovering it from the liquid and placing it on a glass slide. The images in FE-SEM (Figure 1e) show the presence of large oriented aggregates of MOF particles, typically in a face-to-face fashion junction. Those large aggregates have not been previously observed in the solid of the dried MOF at 80 °C after washing (Figure 1c). We speculate that those aggregates are at least preformed during the second stage of the process, when the particles at the bottom of the vessel rise to the top, entrapping some individual MOF particles along the liquid in their rising way to the top to form the floating interphase (Figure 1f).

**Figure 2:**
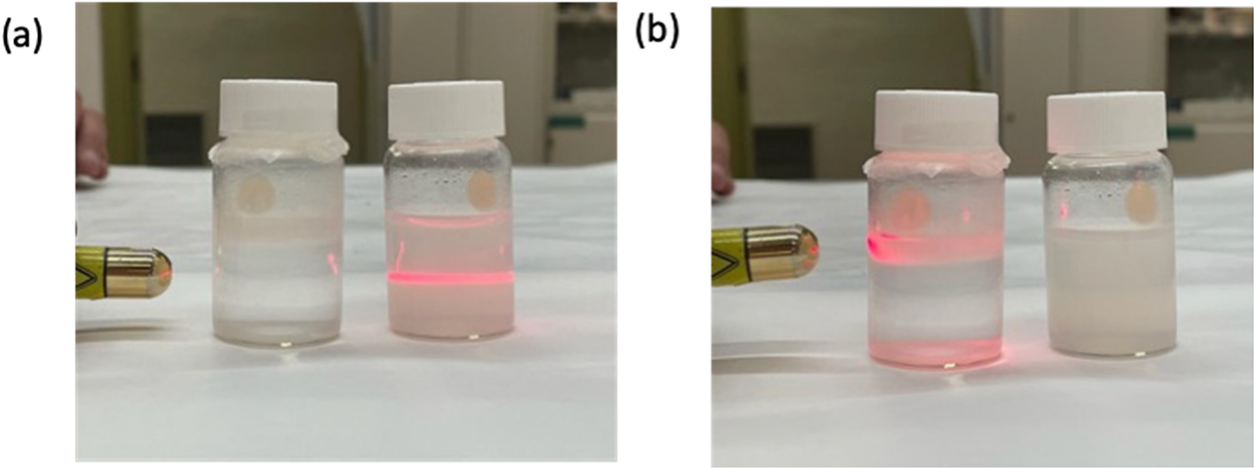
Photography of the laser trace coming across the floating film (left vial) and colloidal dispersion of ZIF-8 nanoparticles (right vial) pointing below the film (a) and at the film (b).

Once this particular phenomenon was observed, the next step to be afforded is the role of the crystal morphology in the phenomena as each morphology will be expected to expose at their surfaces and edges several differences in terms of chemical composition,^15^ for example, cubes are expected to be more enriched in Zn metal modes than RD at the crystal surface, which would be intuitively counterbalanced by counterions present in the synthesis media. Figure 3 shows that the floating interphase is achieved when all ZIF-8 crystal morphologies prepared in the current work are used, i.e., particles with rhombic dodecahedra (RD), cubic particles (CB), and truncated rhombic dodecahedra (TRD) morphologies (Figure S3). Each redispersion of the same amount (1 mg mL^–1^) of quasi-cube crystals or ZIF-8 TRD in water forms a floating phase on top of water after slightly shorter time than the one required for RD ZIF-8, 24–96 h. Thus, even if the surface chemistry of each morphology can be slightly different, those variations are not enough to disable the phenomena.

**Figure 3:**
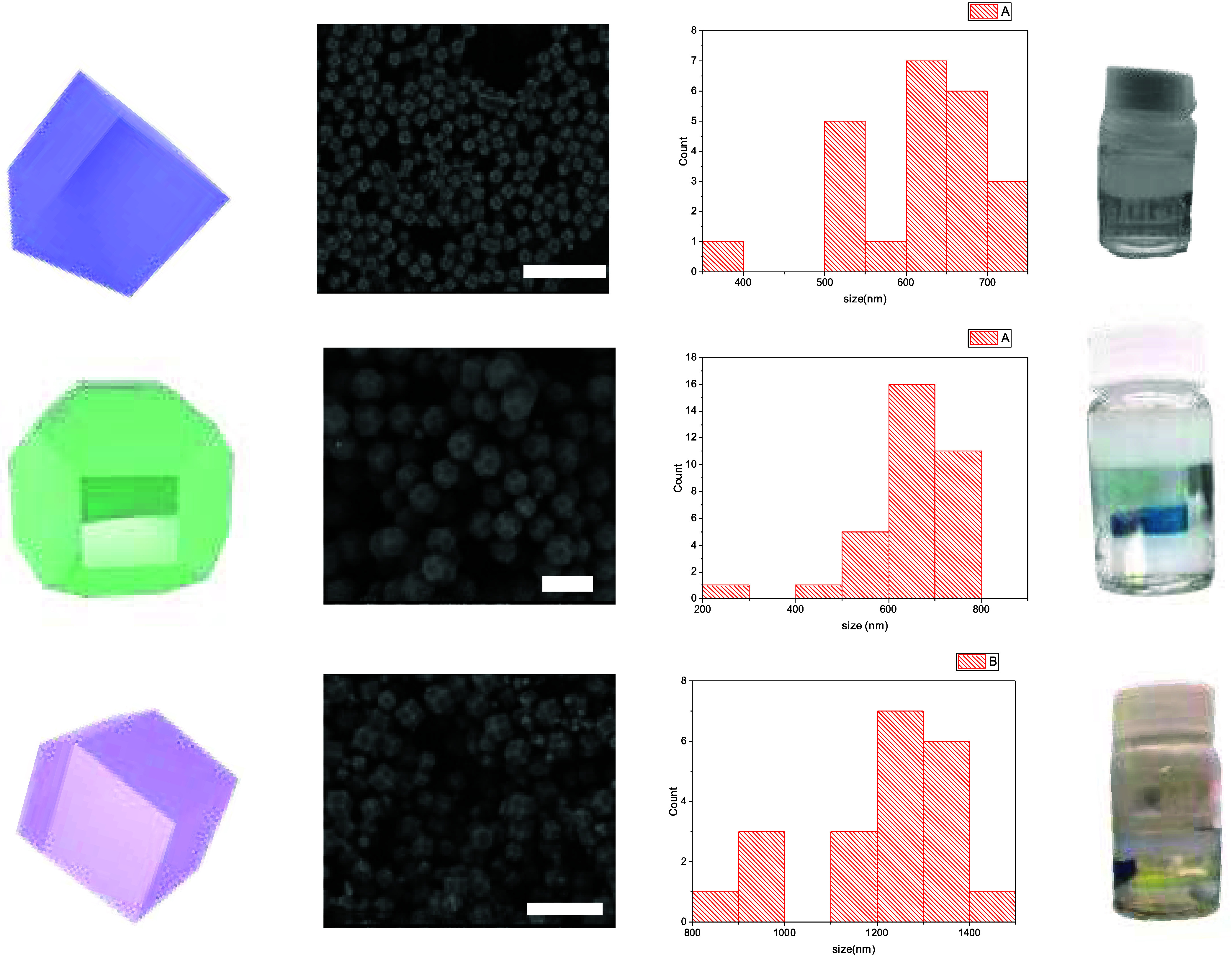
Morphology scheme of ZIF-8, FE-SEM images, particle size distribution, and film images (scale bar: 3, 1, and 3 μm, respectively).

The permanent porosity of the different morphologies and crystal sizes is determined by exposing the dried solid to nitrogen adsorption, and data are integrated on the BET equation, giving the typical values expected for typical ZIF-8 structure (Table S1).

It must be noted that varying the synthetic conditions, mainly time and/or concentration of the starting reagents, several particle sizes of ZIF-8 are synthetically accessible.^15^ Then, ZIF-8 of different particle sizes was prepared, pursuing the range of formation of the floating film. Results show that the particle size must be above 500 nm in diameter. Smaller particles, like 100 nm, provide a stable suspension of ZIF-8 in water (Figure S4) that remains stable over more than 6 months. This observation supports the hypothesis regarding the need for coalescence and aggregation in the film formation.

Another observation on the system under study is that after a certain time (typically more than 2 months), some films evolved, showing the presence of solid at the bottom of the vials, increasing its quantity over time (Figure S5a). XRD of the extracted and dried bottom solid (Figure S5b) in addition to remaining ZIF-8 phase shows the presence of β-Zn (OH)_2_,^28^ in addition to remaining ZIF-8 phase. The images of those precipitated solids point to a partial decomposition of some of the MOF particles, which strongly indicates that the integrity of the MOF is a necessary condition in the formation and prevalence of the film and its floatability.

Another relevant observation is depicted by comparing FT-IR spectra of the film phase and the fallen solid, in which FE-SEM images pointed to the presence of some amorphous regions (Figure S5c–e). Nonetheless, FT-IR spectra of the solid and particles present at the bottom (Figure S6) do not show relevant differences from typical ZIF-8 materials reported elsewhere;^29^ meanwhile, the spectrum of MOF particles recovered directly from the floating film shows some additional bands. Especially relevant are the observation of a new band centered at 900 cm^–1^ and the change in bands in the region between 2900 and 3100 cm^–1^. Those bands are typically associated with the resonance of carboxylate groups and with the sketching of N–H vibrational bands, respectively. The presence of the band related to carboxylate groups in the floating interphase suggests a key role of the counterion of the metal source (Zn acetate) that would remain, at least, at the external surface of the MOF crystals, promoting higher hydrophobicity on the material and favoring its floatability. It cannot be dismissed that the presence of those carboxylic groups and the organic chains contributed to stabilizing the interparticle interactions. The interphase afforded by these counterions would be necessary to promote interactions with imidazole groups in the MOF surface, facilitating the assembly of the particles and the floating film formation.

To fully support this approximation to floatability, ZIF-8 particles were prepared varying the counterions of the Zn source under analogue synthetic conditions, but using as precursors Zn­(NO_3_)_2_ and ZnCl_2_ instead. ZIF-8 formation is confirmed by XRD, verified by a comparable porosity, and particle sizes are over the floatability range (Figures S7–S10, and Table S1). None of those systems present any kind of floatability, which supports the key role of the counterion (acetate) in the phenomena. The suspension prepared with ZIF-8-NO_3_ presents a white precipitate at the bottom after a few hours of dispersion, while ZIF-8-Cl starts to form a colloidal suspension that slowly precipitates (Figure 3). This experiment demonstrates that the acetates as counterion are crucial to achieve the formation of the floating film. To confirm the different nature of the active interphase of the particles, dry particles of each MOF were pelletized to measure wettability. The contact angle measurement shows a higher hydrophobicity of ZIF-8 (102°) compared with that of ZIF-8 NO_3_ (59°), while ZIF-8-Cl presents a certain degree of hydrophilicity (44°) (Figures S11–S13). This trend is also corroborated by measuring the surface charge of each kind of MOF particle by the Z-potential technique (Figure S10b). The ZIF-8 particles synthesized using Zn acetate present a lower surface charge than those of the MOFs prepared using Zn nitrate, and it is almost 3-fold lower than ZIF-8 prepared with ZnCl_2_ as the metal source. Moreover, dynamic water vapor sorption isotherms at 25 °C of ZIF-8 and ZIF-8-NO_3_ show the expected trend for this material. ZIF-8 is a highly hydrophobic solid and does not fulfill its porosity with water.^8^ ZIF-8 synthesized using zinc acetate shows a water uptake at 95% RH even lower than ZIF-8-NO_3_ (Figure S14). These results prompted us to consider the possible contribution to the floatability of an air–liquid interphase in which the air trapped inside the MOF may play a higher role on the apparent hydrophobicity of the structure favoring the formation of the floating film. Thus, the exchange of water and air in the internal channels of ZIF-8 could promote a dynamic change in the system to raise the particle aggregates. Based on this, we could ascribe the stability of air inside the MOF particles as the origin of the mesoscale structure floatability. This characteristic is further validated by the following counter-test: when air is initially removed from the MOF particle by collapse of its crystalline structure, the 3D mesoscale structure no longer floats, as air cannot re-enter the pores nor the liquid solution. This inability to reabsorb air in a liquid environment highlights the crucial role of stable air pockets in maintaining the low density of the MOF particles.

One of the interests in the obtention of floating interphases is their potential use in environmental remediation. To verify that the MOF particles in the floating phase are still adsorbent materials, an organic red dye 7,7-diazaisoindiogo^30^ was used. The molecules added to a colloidal dispersion of the MOF evolved even faster to reformate the floating interphase (Figure 4a). Then, MOF particles were easily recovered, removing the dye from the water media. In the case of methylene blue dye (MB), the film is slowly formed, removing the dye. Interestingly, the film evolved (Figures 4b and S15) and decreased the initial deep blue tone and changed to light violet. Those observations suggest a partial decomposition of the dye during the process. The system was left to evolve under daylight for 10 more days, showing a fully discolored solution and white film comparable to the initial. The analysis of the aqueous phase by HPLC-MS indicates the presence of small organic species that are derived from the methylene blue molecule, confirming the decomposition of the dye, and so a potential catalytic role of ZIF-8 floating films (Figure S16). XRD and BET of the recovered solids after washing show nonsignificant differences from the pristine particles. TEM images also show the resilience of the MOF particles, where ZnO or other general degradation of the particles is not evidenced (Figure S15 and Table S1). Finally, to demonstrate the generalization of the approach, a second MOF was added (ZIF-67) (Figure 4 and Table S1), showing that over the minimum range of floatability, ca. 800 ± 12 nm was prepared and redispersed to form a floating film interphase in less than 24 h (Figure 4c).

**Figure 4:**
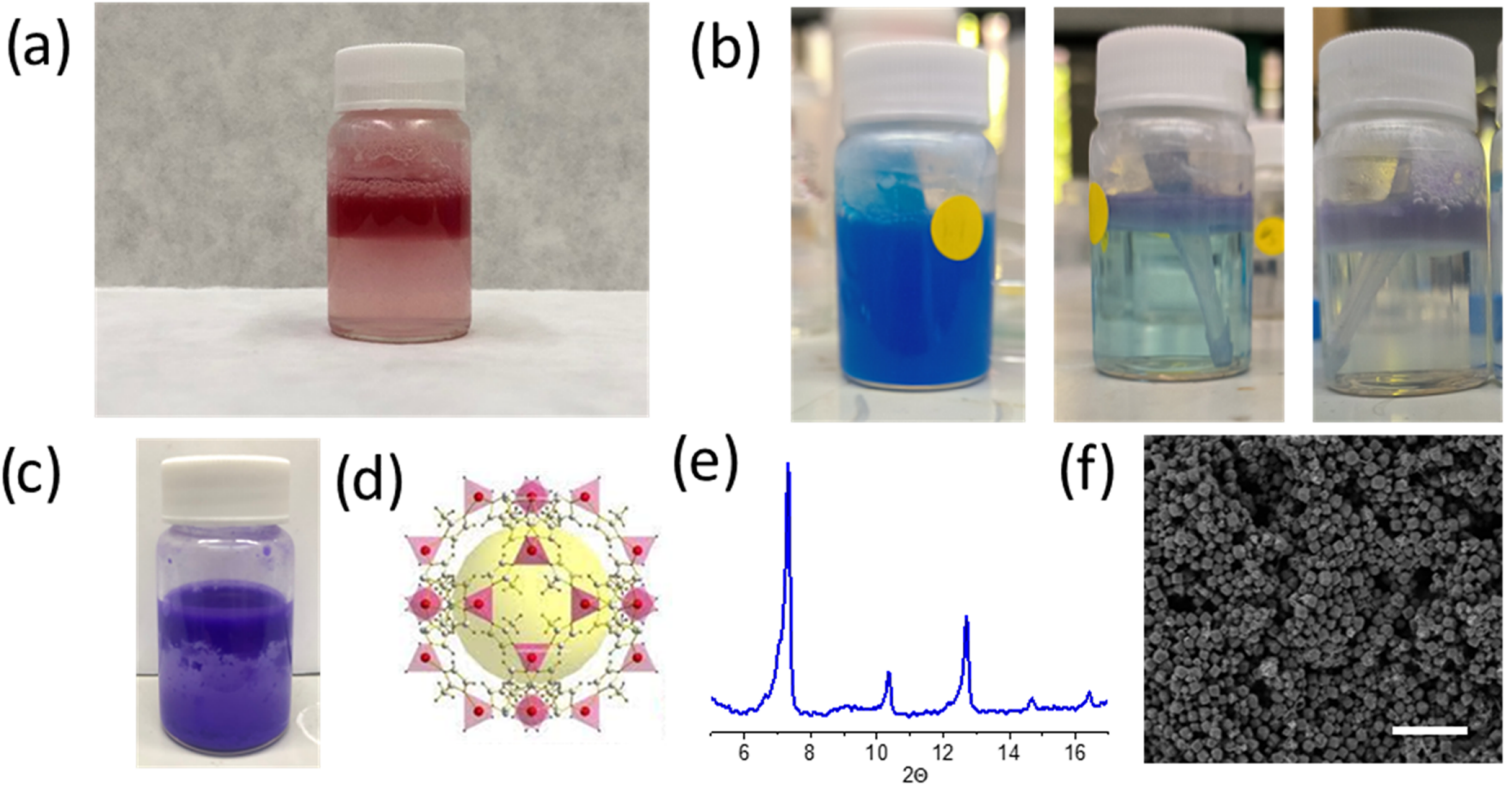
Picture of the floating film containing organic molecules (a) and time evolution of the system (b) exposed to MB at time 0, 5, and 10 days, respectively. Floating film of ZIF-67 (c), scheme of ZIF-67 metal–organic framework (d), PXRD of ZIF-67 (e), and FE-SEM (f) of the dried particles. Scale bar: 5 μm.

## 4 Conclusions

We introduce a new methodology to promote MOF nanoparticles to easily conform as floating interphases between water and air. This method has been applied to the production of ZIF-8 nanoparticles, confirming that the presence of acetate ions and air in the pores of the formed MOF favors the formation of the floating interphase. Moreover, it has been confirmed that the formed MOF particles retain their adsorption capacity while developing discoloration of dye features. We think this is a promising methodology that will allow low energy consumption recovery on separation and remediation systems, fostering the application of nanomaterials at the industrial level. The method has been extended to a second MOF (ZIF-67), which includes redox ions in its structure for the generality of the method. Those MOFs, prepared in water at room temperature, will foster the adaptation of these fascinating nanomaterials to remediation applications.

## Supplementary Material



## Notes

Nitrogen adsorption isotherm and particle size distribution of ZIF-8, X-ray diffractograms of several morphologies, size dependence of the floating phenomena, precited materials image in addition to its X ray diffraction and SEM, IR spectra and BET values of several MOFs, pictures showing ZIF-8, ZIF-8-Cl, and ZIF-8-NO_3_ during the floating process and Z-potential and contact angle of the three MOFs, water adsorption isotherms, floating ZIF-8 interphase after 1 month exposed to MB, and HPLC chromatogram of the solution recovered after 15 days exposed to MB (PDF)
