# Supplementary material for: Self-Formation of Nanoporous Metal–Organic Framework/Water Interphase
Source: ACS Appl Nano Mater. 2026 Mar 31;9(14):6278–84. doi: 10.1021/acsanm.5c05761 (PMC13078347; doi:10.1021/acsanm.5c05761)
Supplement: Supplementary file 1 [file an5c05761_si_001.pdf]

Supporting Information

Self-Formation of Nanoporous Metal-Organic Framework/Water Interphase.

Jaime Gonzalez-Gomez[1], Salvador R.-G. Balestra[2], Alessandro Siria[3,4,5,], Pilar Aranda[1,*], Javier Perez-Carvajal[1,*]*

email: jperez@icmm.csic.es

[1]Instituto de Ciencia de Materiales de Madrid, CSIC, Madrid, 28049, Spain.

[2] Departamento de Física Atómica, Molecular y Nuclear, Área de Física Teórica, Universidad de Sevilla, Sevilla, 41012, Spain.

[3] Laboratoire de Physique de l'Ecole Normale Superieure, ENS, Universit´e PSL, CNRS, Sorbonne Universite, Universite de Paris, Paris, 75005, France.

[4] Center for Advanced Nanoscale Functionalites, college of physics and optoelectronic enginneering Shenzhen Univeristy, Shenzhen, 518060, China.

[5] Department of mechanical engineering, Tsinghua University, Beijing, 100190, China.

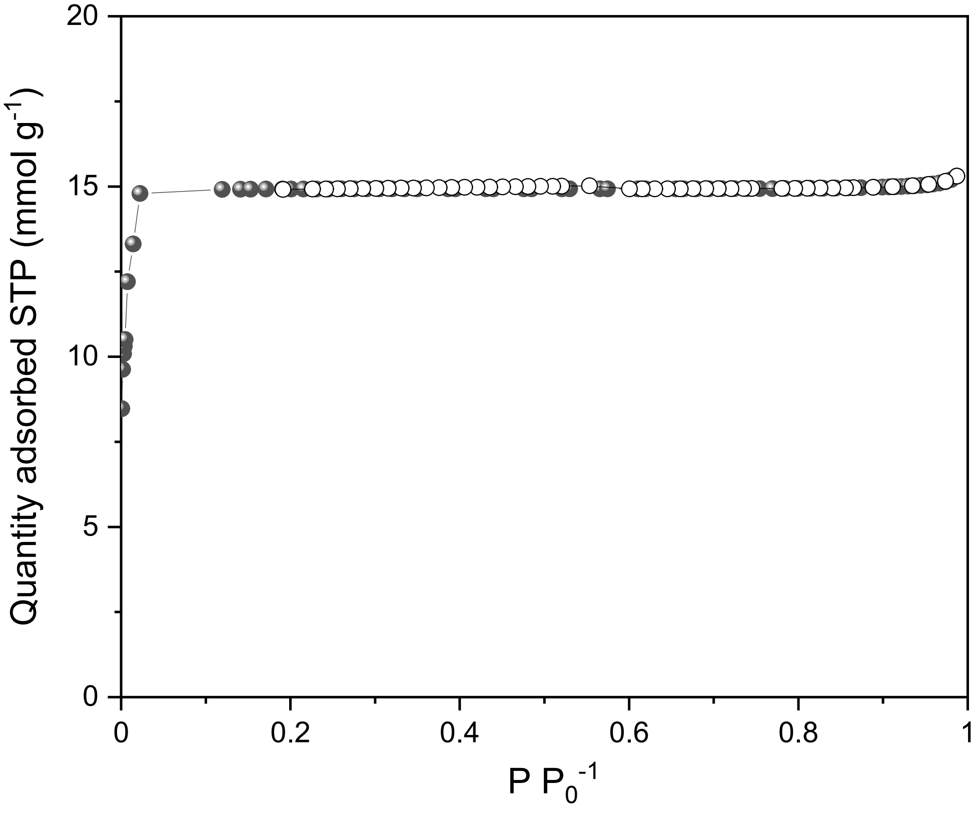

Figure S1. Nitrogen adsorption isotherm of ZIF-8 collected at 77 K

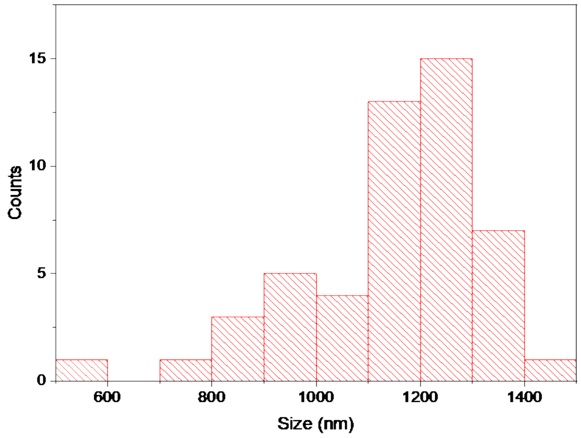

Figure S2. Particle size distrubution of ZIF-8 determined from a FE-SEM images

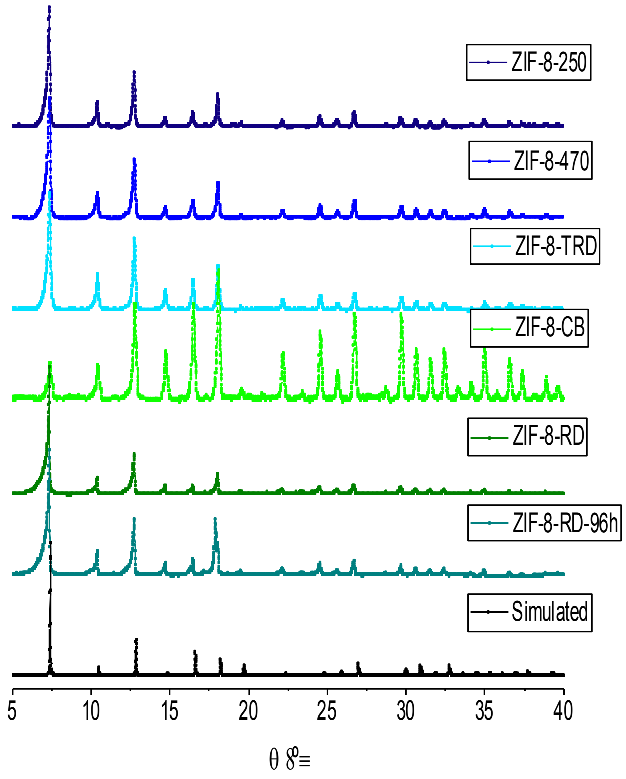

Figure S3. XRD powder pattern of simulated ZIF-8 of the prepared MOF with several morphologies and particle sizes.

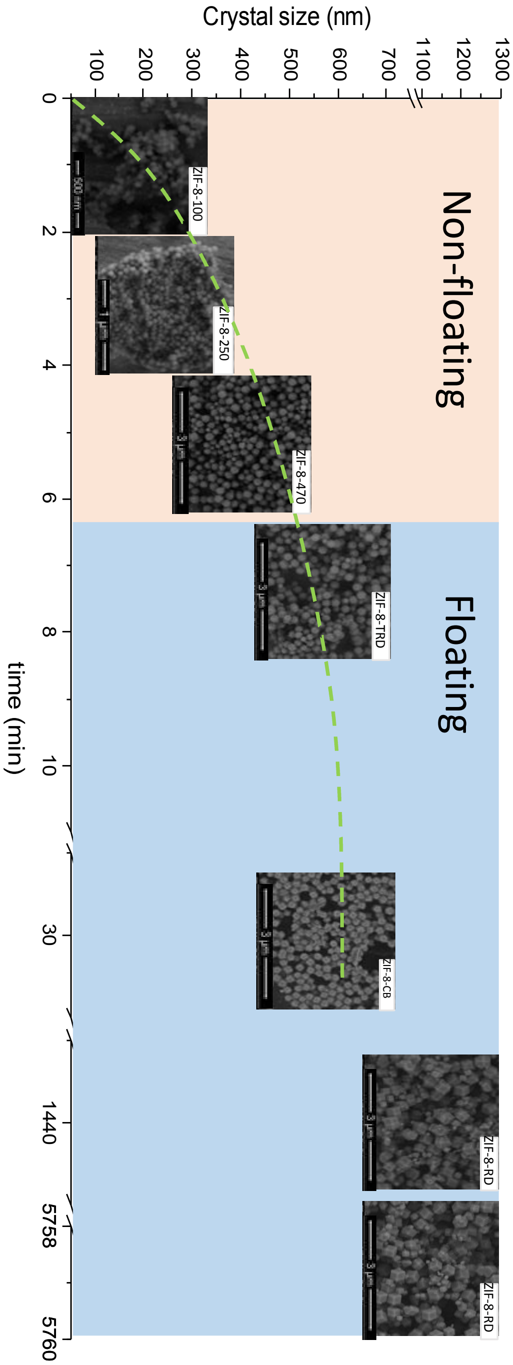

Figure S4. Schemes showing the size dependance of the floating phenomena. Mean size determined from over 10 crystal measurement of size using FEI SEM software.

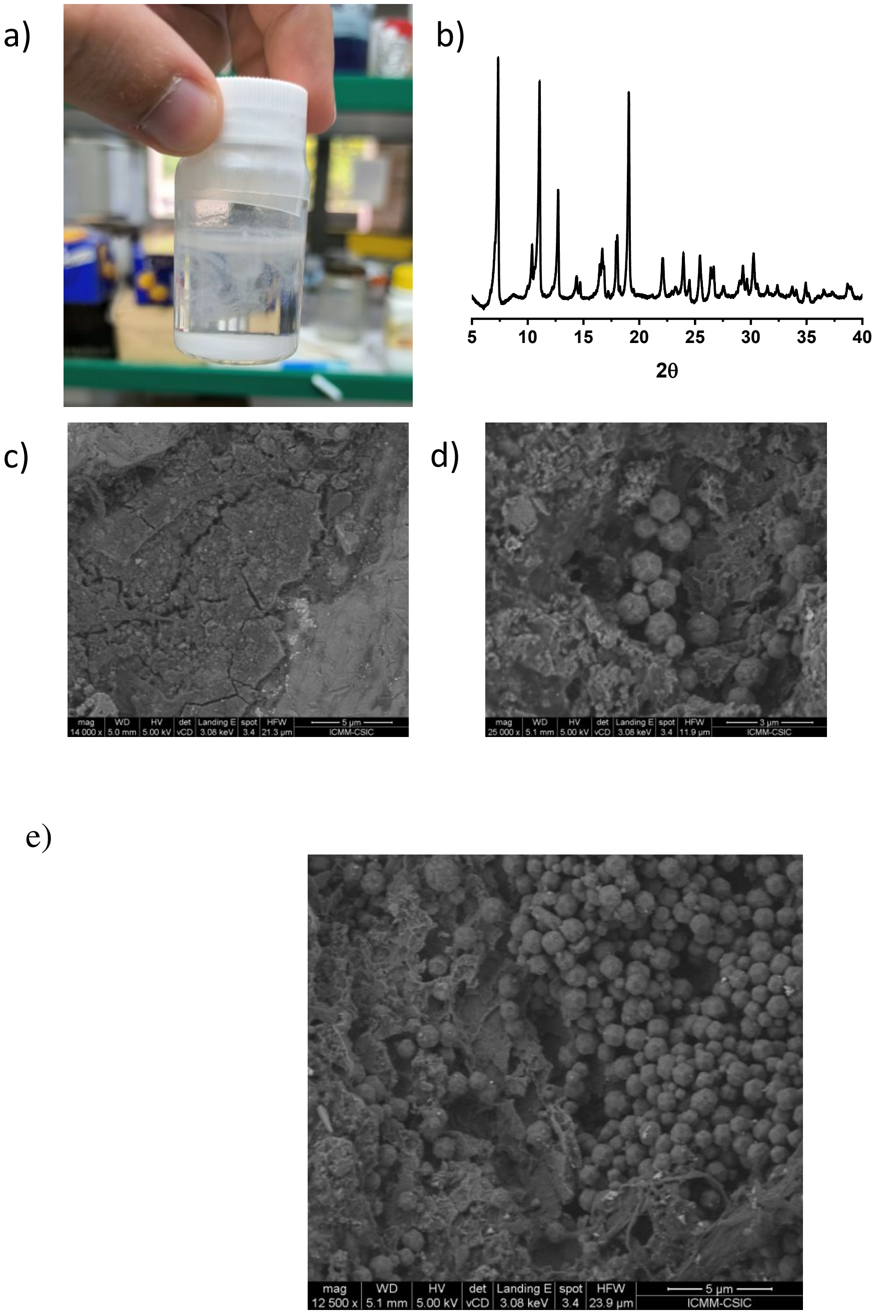

Figure S5. Picture of a ZIF-8 representative sample where it is visible the floating interphase plus a precipitate at the bottom of the vial (a), XRD powder pattern of the precipitated solid (b), and various FE-SEM images of the solid precipitated where it can be observed the presence of inhomogeneities in the sample (c, d and e).

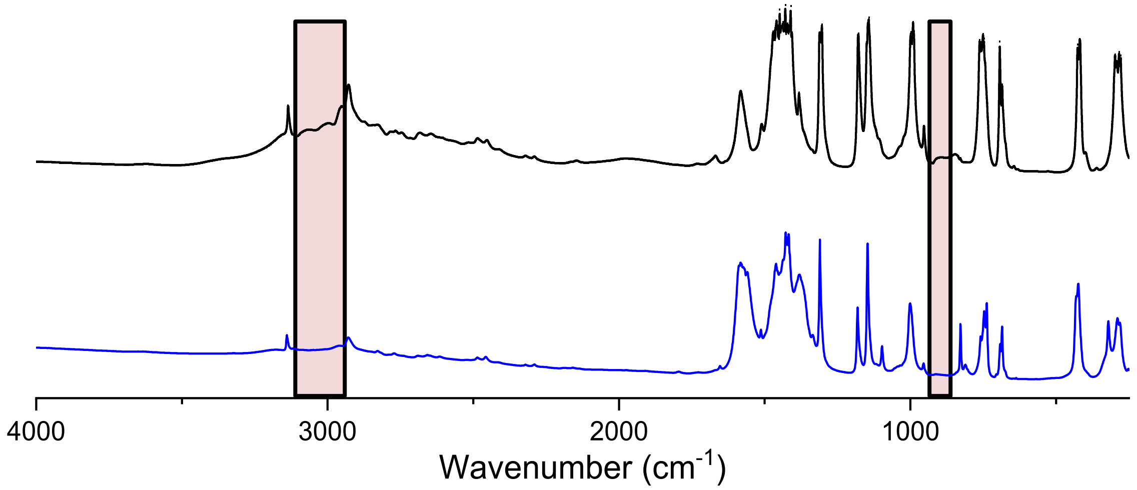

Figure S6. IR spectra of the ZIF-8 precipitated solid (blue) and the ZIF-8 solid collected from the floating interphase.

Table S1. Specific surface area in $m^2\ g^{-1}$ determined by BET method on the nitrogen adsorption isotherms collected from ZIF-8 dried solids (samples submitted to a dynamic nitrogen flux activation at 120°C overnight.

Name	Specific Surface Area
ZIF-8	1299
ZIF-8-CB	1216
ZIF-8-TRD	1308
ZIF8-NO3	1380
ZIF-67	1396
ZIF-8 after MB	1120

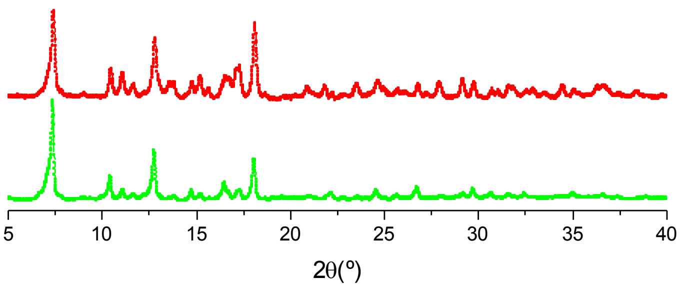

Figure S7. XRD powder difractograms of ZIF-8-Cl (green) and ZIF-8-NO3 (red)

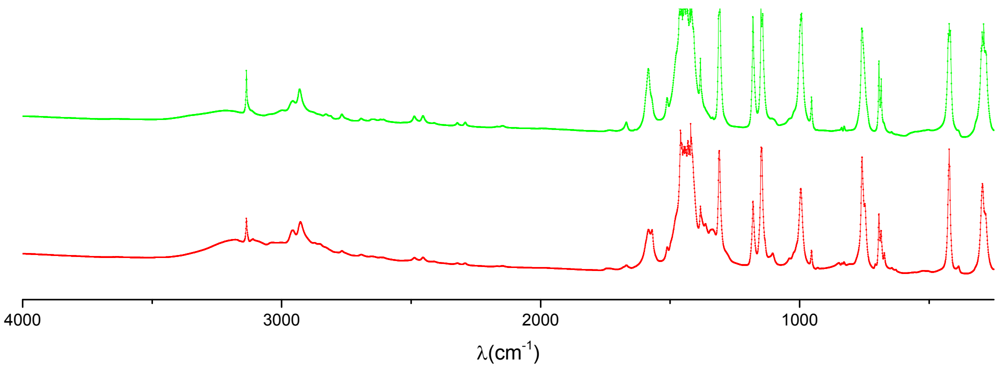

Figure S8. FT-IR spectra of ZIF-8-Cl (green) and ZIF-8-NO3 (red).

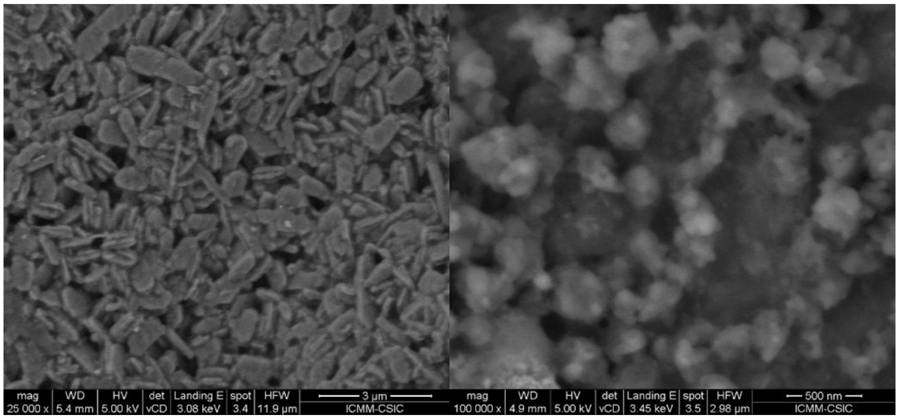

Figure S9- FE-SEM images of ZIF-8-Cl (left) and ZIF-8-NO3 (right)

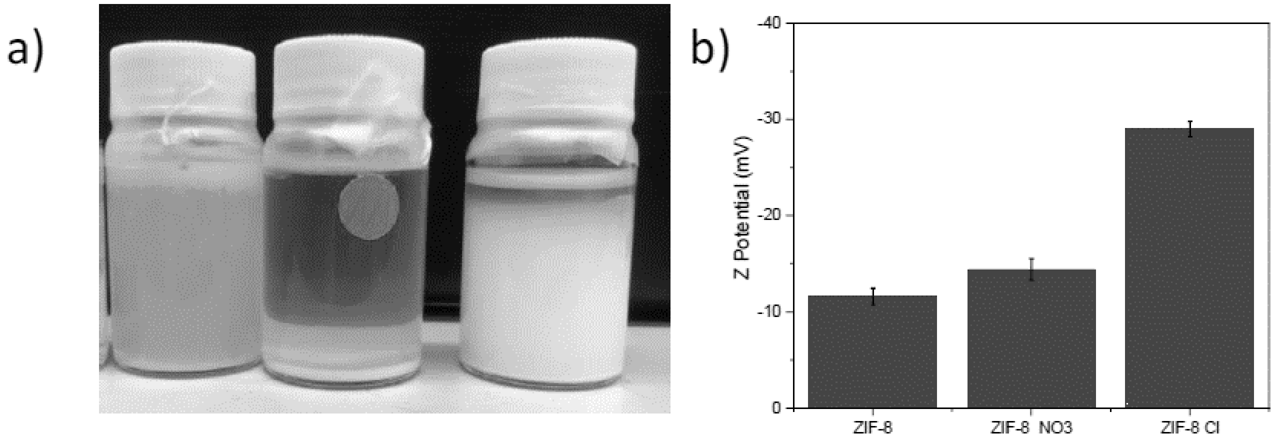

Figure S10. Picture showing from left to right ZIF-8, ZIF-8-Cl and ZIF-8-NO3 during the floating process in addition (a), and Z-potential of the three MOF (b).

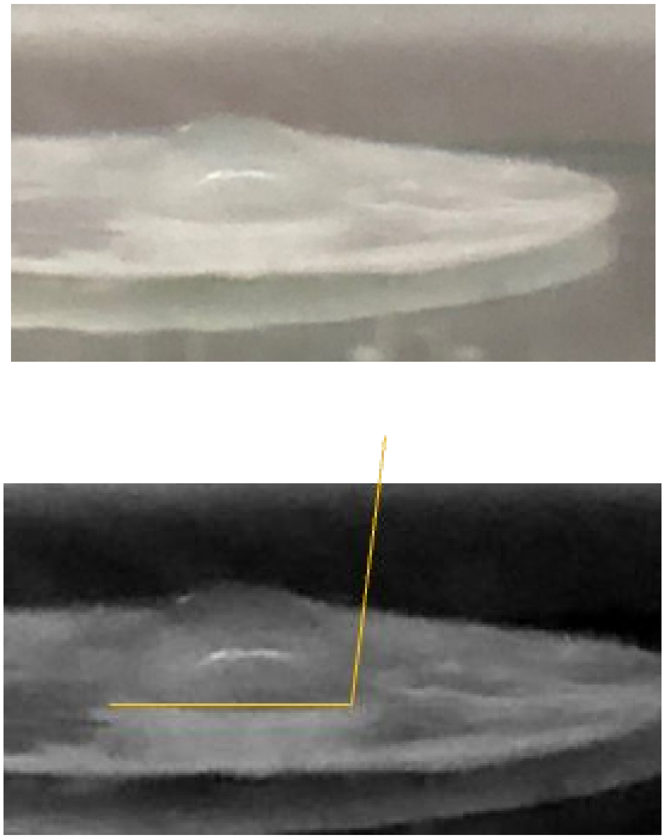

Figure S11. Contact angle picture of ZIF-8 after 1 hour since the water drop is added.

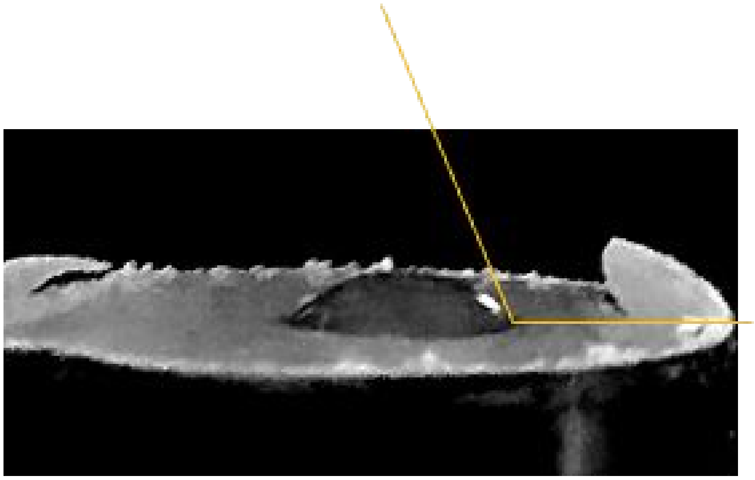

Figure S12. Contact angle picture of ZIF-8-Cl after 1 hour since the water drop is added.

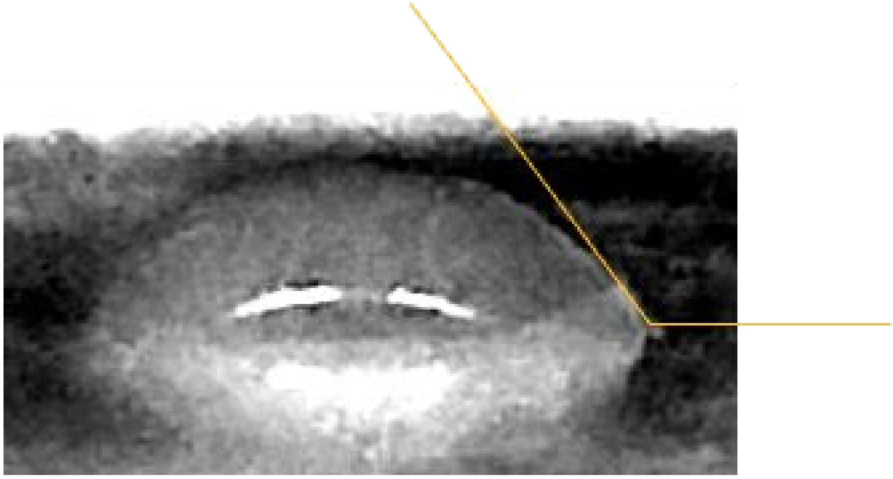

Figure S13. Contact angle picture of ZIF-8-NO3 after 1 hour since the water drop is added.

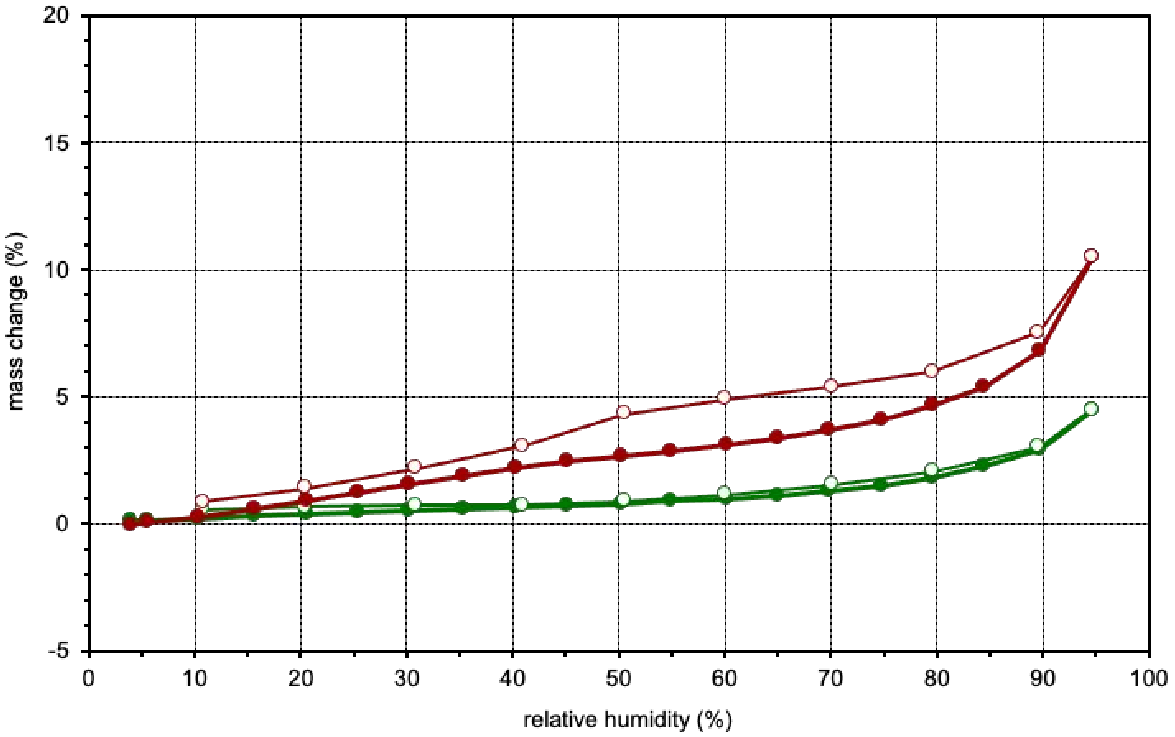

Figure S14. Water sorption-desorption isotherms at 25°C of ZIF-8 (green) and ZIF-8-NO3 (red) where solid dots represent sorption branch at equilibrium and void dots desorption.

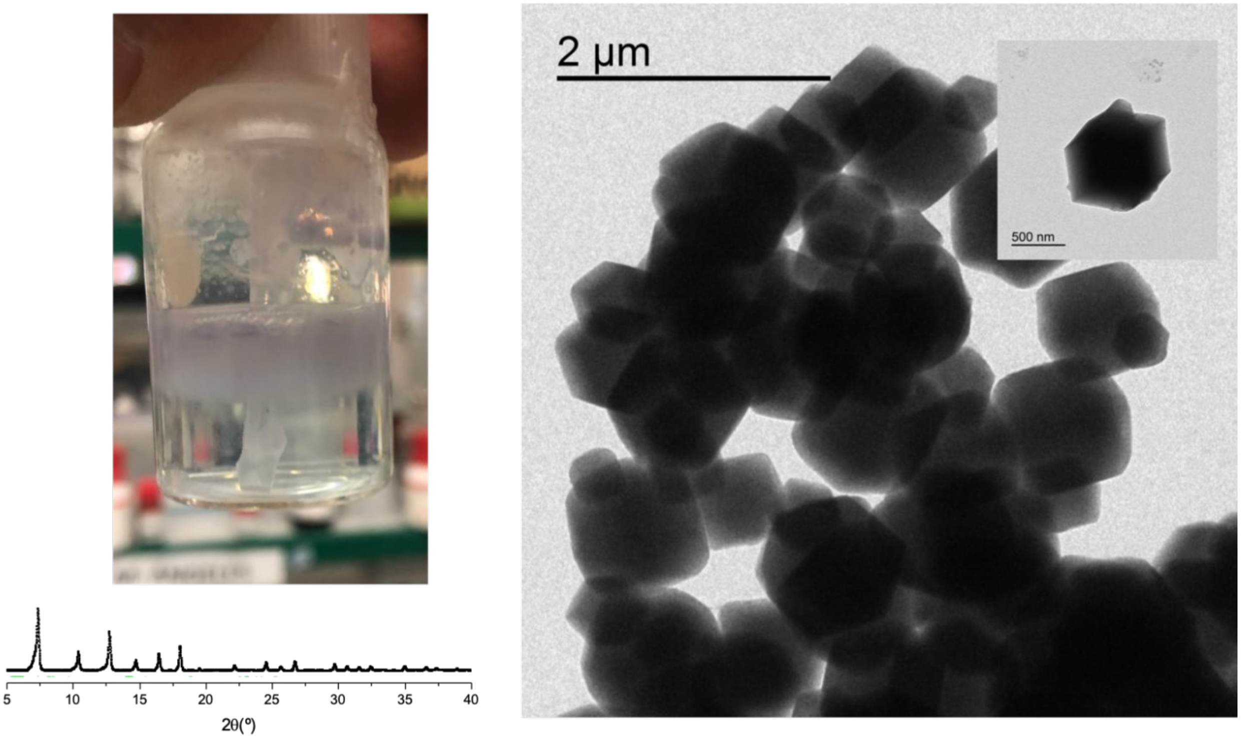

Figure S15. Picture showing the aspect of the floating system after contact with MB for 1 mouth, XRD of the collected solid and TEM image (inset pristine MOF)

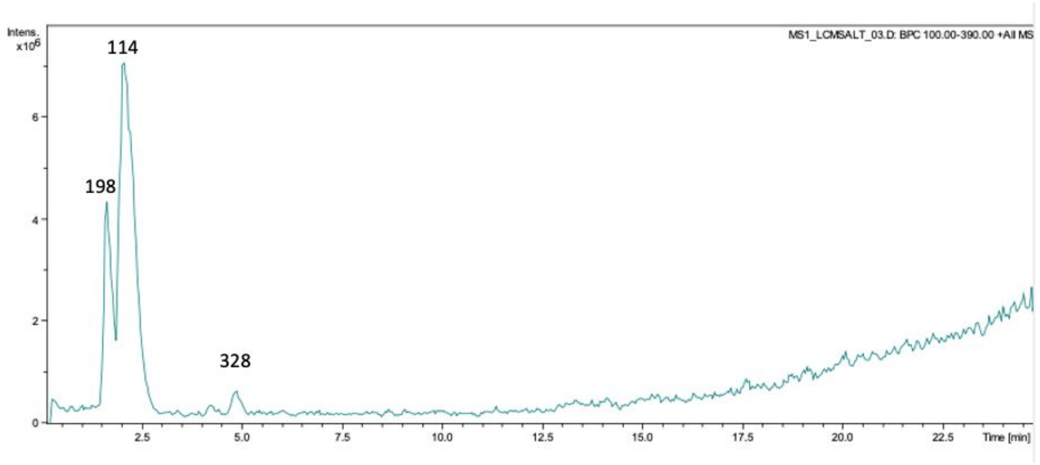

Figure S16. HPLC-MS chromatogram of the solution recovered after 15 days of ZIF-8 in contact with MB dye. Number labeled indicates mass fractions.
